# From Last Resort to Standard of Care: The Evolution and Future of Transcatheter Aortic Valve Implantation

**DOI:** 10.31083/RCM46697

**Published:** 2026-01-21

**Authors:** Oliver Lee, Ahmed Osman, Dominique Shum-Tim

**Affiliations:** ^1^UCL Medical School, University College London, WC1E 6BT London, UK; ^2^Faculté de Medicine, Université de Montréal, Montreal, QC H3T 1J7, Canada; ^3^Division of Cardiac Surgery, McGill University Health Centre, Montreal, QC H3A 1A3, Canada

**Keywords:** transcatheter aortic valve implantation, transcatheter aortic valve replacement, aortic valve stenosis, structural valve degeneration, minimally invasive cardiac surgery

## Abstract

Transcatheter aortic valve implantation (TAVI) has evolved from an experimental, last-resort procedure in 2002 to a first-line therapy for aortic stenosis; moreover, the 2025 ESC/EACTS (European Society of Cardiology/European Association for Cardiothoracic Surgeons) guidelines marked a paradigm shift beyond traditional risk stratification toward earlier intervention and broader patient selection. Current evidence demonstrates the non-inferiority or superiority of TAVI to surgical aortic valve replacement across all risk categories, with the guidelines now recommending TAVI for patients aged ≥70 years and formally endorsing early intervention in asymptomatic severe stenosis when procedural risk is low. Meanwhile, critical challenges persist despite large-scale systematic reviews demonstrating significant mortality reduction following TAVI, including paravalvular leak rates of 10–25% compared to near-zero rates with surgery, and subclinical leaflet thrombosis affecting up to 30% of patients with unclear optimal management strategies. Moreover, the expansion toward younger populations exposes critical knowledge gaps, including unknown long-term durability beyond 10 years, structural valve degeneration rates of 4.8–13.3% at 5–7 years, and complex reintervention scenarios with reported mortality rates of 17.1% for surgical TAVI explantation. Thus, this review synthesizes contemporary evidence within the framework of the 2025 guidelines while examining unique aspects, including the pathophysiology of subclinical leaflet thrombosis, polymeric heart valve technologies as next-generation solutions, and the critical durability questions that will determine the role of TAVI in younger patients. Next-generation polymeric valves utilizing materials such as polyhedral oligomeric silsesquioxanes-polycarbonate urethane (POSS-PCU), poly(styrene-b-isobutylene-b-styrene) (SIBS), and siloxane polyurethane-urea have shown promising preclinical results in terms of enhanced durability and reduced thrombogenicity, although comprehensive clinical validation remains necessary. As TAVI practice evolves under new guideline recommendations emphasizing early intervention and simplified antithrombotic management, this thorough analysis can provide essential context for understanding both current capabilities and future directions in transcatheter valve therapy.

## 1. Introduction

The prevalence of aortic valvular disease, whether through rheumatic heart disease or calcific degeneration, has risen greatly over the last 30 years, with prevalence greatly increased once the individual is aged above 65 years [1]. However, disability-adjusted life years have declined during the same period [2], in part due to advancements in pharmacological management and interventional treatments. Additionally, advances in minimally invasive transcatheter techniques have enabled doctors to offer definitive treatment to millions worldwide and minimize the impact of a debilitating condition.

Transcatheter aortic valve replacement has undergone extensive development to become the safe, effective, and ubiquitous procedure that interventional cardiologists and cardiac surgeons now routinely recommend to patients with stenosed aortic valves. Transcatheter valve technology initially began in the 1990s with balloon valvuloplasty of the pulmonary and mitral valves [3, 4, 5, 6], owing to the lower-pressure system. The focus was later expanded to include the aortic valve to treat symptoms in high-risk patients with aortic stenosis [7]. This was later followed by efforts to replace the valve entirely, led by Cribier and his team in Rouen, France [8]. Indeed, the first percutaneous replacement of a calcified aortic valve through transcatheter technologies was performed by Cribier and his team in 2002 [8]. Notably, the patient suffered from various chronic and debilitating conditions and had frequent episodes of life-threatening cardiogenic shock and was consequently not a candidate for surgical intervention. Thus, transcatheter aortic valve implantation (TAVI) was initially performed as a last-resort effort to potentially bridge to surgical implantation. No acute episodes of heart failure were identified during the post-procedure period, and the valve leaflets remained mobile, with no signs of regurgitation. Unfortunately, the patient died 17 weeks after the procedure, after suffering from several noncardiac complications such as pulmonary embolism and sepsis. Despite this, the operation served as a landmark proof of concept and spurred several feasibility studies to evaluate whether this technology could potentially replace surgical intervention, particularly in high-risk patients who could not tolerate the invasive procedure.

As TAVI has evolved over the years and further clinical trials are conducted, the patient population amenable to TAVI continues to expand. Indeed, the recent publication of the 2025 ESC/EACTS (European Society of Cardiology/European Association for Cardiothoracic Surgeons) guidelines [9] represents a significant paradigm shift, recommending TAVI rather than traditional risk-based approaches, even in asymptomatic patients. This represents a notable departure from the historical “watchful waiting” approach and incorporates evidence from trials such as EARLY transcatheter aortic valve replacement (TAVR), thereby demonstrating how TAVI technology has matured. However, this expansion into younger, lower-risk populations amplifies critical knowledge gaps inadequately addressed in the existing literature. While previous reviews have primarily focused on established high-risk populations, this comprehensive analysis examines TAVI while addressing two critical frontiers: the emerging challenge of subclinical leaflet thrombosis affecting up to 30% of patients, with unclear optimal management strategies, and the promise of next-generation polymeric heart valve technologies that may fundamentally address current limitations.

## 2. The TAVI Procedure

### 2.1 Indications

The 2025 ESC/EACTS guidelines [9] have significantly updated the indications for TAVI, representing the most contemporary evidence-based recommendations available. The American guidelines remain based on the 2020 American College of Cardiology/American Heart Association (ACC/AHA) recommendations [10]. These guidelines incorporate robust evidence from recent trials and mark a fundamental shift toward earlier intervention strategies. Nonetheless, while these can help inform clinicians whether a patient may receive net benefit from a TAVI procedure, individual decisions may be influenced by a variety of clinical and non-clinical factors, including patient anatomy, operator experience, and patient preference. Both guidelines state a class 1 recommendation of TAVI in patients of advanced age (>65), lower life expectancies, and higher risk scores. Further details are elucidated in Table 1 (Ref. [9, 10]).

**Table 1.** **A comparison of the 2020 ACC/AHA and 2025 ESC/EACTS guidelines**.

Patient population	2020 ACC/AHA [10]	2025 ESC/EACTS [9]
Age: >80 years	TAVI recommended (class 1A)	TAVI recommended (class 1A)
Age: 65–80 years	TAVI recommended with shared decision-making (class IA)	SAVR or TAVI based on individual patient characteristics (class IB)
Age: <65 years	SAVR preferred, especially if life expectancy >20 years	SAVR preferred if low surgical risk (STS-PROM <4%)
Asymptomatic severe aortic stenosis	Not specifically addressed	Early intervention recommended if low procedural risk (class IIa A)
High STS: ~8%	TAVI recommended regardless of age	TAVI recommended (STS-PROM/EuroSCORE >8%) (class IA)
Low STS: ~4%	Age-based approach with shared decision-making	SAVR preferred in patients <75 years
Prohibitive risk	TAVI is only viable option (class IA)	TAVI recommended for inoperable patients (class IA)

ACC, American College of Cardiology; AHA, American Heart Association; EACTS, European Association for Cardiothoracic Surgeons; ESC, European Society of Cardiology; EuroSCORE, European System for Cardiac Operative Risk Evaluation; SAVR, surgical aortic valve replacement; STS, Society of Thoracic Surgeons; STS-PROM, Society of Thoracic Surgeons Predicted Risk of Mortality; TAVI, transcatheter aortic valve implantation.

### 2.2 Access and Procedure

Cribier’s landmark procedure was performed through access via the right femoral vein, requiring a transseptal puncture to access the left ventricle [6]. Today, this method has been replaced by direct arterial access through either the femoral or subclavian artery [11], or more rarely, through direct aortic puncture. More than 80% of cases adopt a femoral approach [12]; however, this approach requires favourable peripheral arteries with minimal tortuosity and calcification, and, thus, may not be suitable for patients with severe peripheral artery disease or abnormal anatomy [13]. Therefore, alternative arterial access routes have been developed and are gaining increasing attention. The transaxillary approach offers a viable option for patients with prohibitive iliofemoral anatomy [14]. The transaxillary route offers several advantages due to the associated lower calcification profile, proximity to the heart, and large-vessel diameters typically greater than 6 mm [15]. Current ESC/EACTS [9] guidelines classify transaxillary access as class IIb for patients deemed unsuitable for transfemoral access. Recent propensity-matched analyses have demonstrated similar 30-day mortality rates (5% vs. 6%) when compared to the standard transfemoral approach [16]. However, patients undergoing transaxillary procedures were noted to be more likely to suffer from myocardial infarctions (4% Tax (transaxillary) vs. 1% TF (Transfemoral)). This was attributed to patient selection factors. Five of the eight patients who suffered from periprocedural myocardial infarctions had previous coronary artery bypass grafting (CABG) utilizing the left internal mammary artery (LIMA) as a graft to the left anterior descending artery (LAD). As the TAVI sheath was advanced past the LIMA ostium, ischemia was induced, resulting in hemodynamic instability or ventricular fibrillation. The authors recommend that a patent LIMA graft be considered a contraindication to left axillary access in TAVI. Despite this, larger-scale studies still demonstrate excellent outcomes: a systematic review of 1519 patients by Herremans *et al*. [16] reported a procedural success rate of 92.2% and a 30-day mortality of 5.2%. Techniques to access the apex of the heart via a mini-thoracotomy have also been developed, but remain more invasive and possess higher risks of mortality [17, 18].

As mentioned, the aortic valve is usually accessed through a retrograde approach via a standard Seldinger technique [19]. Venous access is simultaneously obtained for right ventricular pacing [12]. The patient is then anticoagulated with unfractionated heparin or bivalirudin [20].

The aortic valve is crossed using a guide wire, following which balloon aortic valvuloplasty (BAV) is performed. Here, a balloon is inflated across the aortic valve to compress the calcified aortic valve against the vessel wall, dilate the aortic annulus, and make room for subsequent deployment of the prosthetic valve. Subsequently, the balloon is retracted and swapped for the TAVI prosthetic. The aortic valve gradient and flow across the valve are then assessed using fluoroscopy or transoesophageal echocardiography (TOE). The catheters are withdrawn, and vascular access sites are closed. The patients are then prescribed either a combination of clopidogrel or aspirin; however, the choice of anticoagulant is largely center-dependent.

During deployment of BAV and aortic valve prosthesis, left ventricle (LV) outflow is iatrogenically reduced to decrease the risk of balloon dislodgement from the outflow tract, balloon rupture, or valve embolism. This is commonly achieved by rapid ventricular pacing at 160–220 beats per minute or by inducing ventricular asystole with adenosine administration [12].

### 2.3 Imaging

Preprocedural and periprocedural imaging is tantamount to the success of the procedure [21]. Undersizing may result in insufficient radial force on the valve after deployment, leading to valvular embolism or paravalvular leaking [22, 23], whereas oversizing may result in coronary obstruction, conduction abnormalities, or annular rupture [24, 25, 26].

Regarding coronary assessment, the new 2025 ESC guidelines formally endorse a computed tomography (CT) first approach and recommend class 1B cardiac CT angiography (CCTA) for patients with a <50% likelihood of obstructive coronary artery disease [9]. If obstructive coronary artery disease is identified, angiography is then performed, informing the need for pre- or periprocedural stenting of the coronaries. This approach avoids invasive imaging in patients with a low likelihood of coronary artery disease.

To assess the aortic annulus, both TOE and CT are used. However, echocardiography has been shown to underestimate the annular size in about half of patients [27, 28], which may be explained by the fact that CT provides higher spatial resolution than two-dimensional (2D) echocardiography. Despite this, both measures demonstrate satisfactory clinical outcomes. Cardiac magnetic resonance imaging (MRI) has also been used in imaging trials to assess the anatomic environment of the aortic valve; however, there is a distinct lack of data on the effectiveness of this technique in TAVI [29].

### 2.4 Postoperative Care and Common Complications

Appropriate postprocedure monitoring and management are important to ensuring consistent and favourable patient outcomes across all populations [30]. As mentioned previously, conduction disturbances are a relatively prevalent complication of TAVI procedures. Radial force and compression of the left ventricular outflow tract (LVOT) during valve deployment can disrupt the delicate fibers of the atrioventricular (AV) node and bundle of His [31]. To surveil for this, patients require continuous electrocardiogram (ECG) monitoring. However, almost two-thirds of patients with complete AV block recover normal conduction [32]. Nonetheless, using a pacemaker remains critical for unresolved conduction abnormalities, and implantation rates range from 5% to 40% [33]. Additionally, electrolyte balance, water balance, and renal function are important parameters to monitor postoperatively due to the risk of contrast-induced nephropathy, which is most prevalent within the first 72 hours after contrast administration [34].

Notably, despite advances in TAVI technology, such as cerebral embolic protection (CEP) devices, stroke remains a major risk after TAVI [35]. Indeed, stroke rates remain around 4–6% in patients deemed high risk, only decreasing to around 3% [36] in more recent trials with low-risk patients [37]. Due to the action of passing a calcified aortic valve with a catheter, calcific embolic fragments can cause intracranial lesions, with subclinical lesions being detected on diffusion-weighted (DW) MRI in 68–84% of patients. Some studies have found stroke to be more common post-TAVI than in surgical aortic valve replacement (SAVR) [38, 39] and, as such, antithrombotic regimens are commonly prescribed to patients post-procedure. The new 2025 guidelines recommend single antiplatelet therapy after TAVI, rather than dual antiplatelet therapy (DAPT), following a trial that found that the incidence of bleeding and the composite of bleeding or thromboembolic events was lower with aspirin than with DAPT at 1 year [40]. However, the specific regimen should be tailored based on patient characteristics and co-morbidities [41] as the recommendation is for patients with no other oral anticoagulant (OAC) indication.

Another common complication within the realm of TAVI is injury to the arterial vasculature or to the site of access. Indeed, the larger the catheter, the more likely the procedure is to cause various vascular pathologies [42]. Additionally, risk factors such as peripheral vascular disease and female gender may also increase the likelihood of vascular injury [42, 43]. As the catheter is passed over the delivery sheath, the catheter may contribute to the formation of a hematoma, aneurysm, or dissection. Furthermore, vessel perforation or embolization may occur while the catheter is advanced toward the aorta. Vascular injury has been shown to increase mortality up to 3 times [44]. The PARTNER B trial [44] performed in 2010 found that the rate of vascular complications within 30 days of TAVI was 16%.

### 2.5 Complications Unique to TAVI

While prone to similar complications involved in other cardiac surgeries, such as stroke, conduction disturbance, and infection, TAVI also includes various unique complications. One of the more notable complications is a rupture of the aortic annulus, with studies reporting almost a 7-fold increase in 30-day mortality (49–67%) when compared to those without rupture [45]. Here, as the valve is expanded, excessive radial force may fracture any weakened tissue, rupturing the aortic annulus. This is far more likely in balloon-expandable valves (BEVs) [46]. Key indicators for when a patient may be at risk of annular rupture have been extensively studied. Several authors have purported that subannular calcifications play a major role in annular rupture [47], with an almost 11-fold increase (odds ratio (OR): 10.92; 95% confidence interval (CI): 3.23–36.91; *p* < 0.001). Additional risk factors include a smaller body surface area and aggressive postdilatation. Meanwhile, valve oversizing has also been identified as a strong predictor of annular rupture [48]. Thus, if a patient has been identified as at high risk of annular rupture, careful consideration of valve sizing and valve type should be given to reduce the risk of rupture.

Prosthesis embolism has also been identified as a unique complication, defined as the movement of the prosthesis to the degree that the prosthesis loses contact with the annulus. The resulting consequences can be drastic, potentially causing severe hemodynamic collapse through acute aortic regurgitation or mitral valve obstruction, obstruction of the coronaries or ascending aorta, or stroke if the valve fragments are dislodged [49]. Initially, the first generations of self-expanding valves (SEVs) were known to be independent predictors of embolization; however, current generations exhibit excellent rates of valve embolization [50]. Interestingly, and in contrast to aortic rupture, calcification around the annulus is inversely related to the rate of embolization. Thus, authors have theorized that calcium aids in anchoring the valve in place, and without it, the valve lacks support [49]. Bicuspid valves have also been shown to be independent predictors of embolization [51]. This may be due to a multitude of factors, including unclear borders between the annulus and leaflet, the larger annulus dimensions in bicuspid patients, and the non-tubular nature of the AV complex in these patients. Rates of embolization can be reduced in these patients by considering supra-annular deployment or slightly oversizing the valves to aid valve seating [51]. Treatment of valve embolization typically depends on the location of the embolized valve. Management normally involves open surgical retrieval [52]; however, in some cases, valves may be salvaged through valve-in-valve (ViV) anchoring [53].

## 3. Evolution of Risk Stratification

### 3.1 High-Risk Patients: The Pioneer Studies

Long before the development of the current guidelines, in which TAVI is widely recommended to older patients who may be less tolerant of SAVR, TAVI was previously reserved for patients who were unable to tolerate surgery. In relatively healthy patients, SAVR was associated with low operative mortality and improved symptoms. However, in 2010, around 30% [44] of patients with severe aortic stenosis requiring valve replacement were unable to undergo surgery due to comorbidities or advanced age. The paucity of treatment options for high surgical-risk patients necessitated numerous trials investigating the safety and efficacy of TAVI across varying surgical risk scores. The PARTNER1 [44] trial was a landmark study, one of the first large-scale randomized controlled trials (RCTs) conducted for high-risk patients, recruiting 360 patients internationally (the majority of whom were US-based). After 1 year, death from any cause was 30.7% in the TAVI population and 50.7% in the SAVR population (hazard ratio (HR): 0.55; 95% CI: 0.4–0.7; *p* < 0.001). However, strokes were more common within the TAVI group, a trend that has remained consistent over recent years [54]. Despite operator inexperience and immature device technology, which may have contributed to postoperative complications, the PARTNER1 trial [44] demonstrated that TAVI had the potential to be the new standard of care for high-risk patients requiring aortic valve replacement.

### 3.2 Intermediate-Risk Evidence

Once the efficacy of TAVI for high-risk patients was demonstrated, centers around the world began to utilize the technique in intermediate or low-risk patients. To substantiate this shift in application, rigorous evidence would have to be collected on the usage of TAVI in this cohort. Thus, the PARTNER2 [55] and SURTAVI [56] trials enrolled a large number of patients within the upper and lower limits of the Society of Thoracic Surgeons (STS) scores. Both trials found that TAVI was non-inferior to SAVR after 2 years. However, TAVI was associated with higher rates of stroke and need for pacemaker implantation, yet also provided larger aortic valve areas and improved valvular hemodynamics. Conversely, SAVR was associated with higher risks of acute kidney injury (AKI), atrial fibrillation, and transfusion requirements. Notably, both trials used different forms of TAVR: PARTNER2 used BEVs, while SURTAVI utilized SEVs.

### 3.3 Low-Risk Paradigm Shift

These trials were later followed by RCTs investigating TAVI outcomes in low-risk patients, completing the study of TAVI across the whole spectrum of surgical risk scores. TAVI, being the preferred method for aortic valve replacement in low-risk patients, would represent a massive paradigm shift within cardiac surgery and interventional cardiology, and, as a result, multiple trials were conducted to validate this shift [57, 58]. The PARTNER3 trial [59] found that at 2 years, TAVI was associated with lower risks of death from any cause (TAVI: 11.5%, SAVR: 17.4%; HR: 0.63; 95% CI: 0.45–0.88; *p* = 0.007). The NOTION trial [57] found no statistically significant difference in all-cause mortality (TAVI: 65.5%, SAVR: 65.5%; HR: 1.0; 95% CI: 0.7–1.3; *p* = 0.9). The EVOLUT low-risk trial [60] found that at 2 years, TAVI was non-inferior to SAVR with respect to all-cause mortality or disabling stroke (TAVI: 4.3%, SAVR: 6.3%; *p* = 0.084). The DEDICATE trial [58] in Germany found that TAVI was associated with improved rates of all-cause death or stroke after 1 year (TAVI: 5.4%, SAVR: 10%). These studies also affirmed that TAVI was associated with improved valve performance, as evidenced by lower transvalvular gradients and higher valve area.

While these RCTs demonstrate similar findings, these studies utilized different transcatheter heart valve (THV) systems (balloon-expandable vs. self-expanding), and, as such, level 1A evidence in the form of meta-analyses was required to support a significant shift in surgical practice. Subsequently, a large-scale systematic review and meta-analysis were performed [61], collecting the studies mentioned above across all surgical risk scores. The review included seven relatively homogenous trials (r^2^ < 0.001) encompassing 8020 patients (4014 TAVI and 4006 SAVR). When pooled, TAVI was associated with a significant decrease in all-cause mortality compared to SAVR (HR: 0.88; 95% CI: 0.78–0.99; *p* = 0.03), with the highest survival benefit in patients undergoing transfemoral TAVI (17% relative risk reduction (RRR)). Subgroup analysis confirmed no difference in mortality when either THV system was used, and both systems demonstrated superiority to SAVR. However, TAVI was found to carry an increased risk of pacemaker-implantation post-procedure, contributing to the 30-day stroke risk post-procedure. Interestingly, as this meta-analysis included patients from all risk categories and identified a reduction in mortality throughout the whole spectrum, the study was crucial in informing the current most recent guidelines from the ACC/EACTS, which suggest that TAVI should be routinely offered to those aged >65 years as opposed to using the STS/EuroSCORE to inform decisions. A brief smmary of the major milestones in TAVI development and their supporting studies has been compiled within Table 2 (Ref. [9, 11, 32, 33, 34, 42, 51, 54, 55, 56, 62, 63, 64, 65, 66, 67, 68, 69, 70, 71]).

**Table 2.** **A summary of major milestones and paradigm shifts in TAVI development**.

Milestone	Key changes	Landmark studies	Impact on practice	References
First-generation TAVI (2002–2011)	Introduction of balloon-expandable (Sapien) and self-expanding (CoreValve) valves	PARTNER 1A/1B	Established TAVI for inoperable/high-risk patients; 1-year mortality reduced by 20% vs. medical therapy	[42, 51]
Intermediate-risk expansion (2016)	TAVI approved for an STS score of 4–8%	PARTNER 2, SURTAVI	Non-inferiority to SAVR demonstrated; 2-year mortality ~16–17%	[62, 63]
Low-risk paradigm shift (2019)	TAVI for patients with an STS <4%	PARTNER 3, Evolut Low Risk	Superior to SAVR at 1 year (composite endpoint 8.5% vs. 15.1%); lower stroke rates	[54, 55, 56]
Asymptomatic severe AS (2024)	Early intervention before symptom onset	EARLY TAVR, AVATAR	50% reduction in death/stroke/hospitalization; challenges “watchful waiting” approach	[9, 64]
Next-generation valves	Repositionable/recapturable systems; external sealing skirts; 14F delivery systems	Multiple iterative studies	Paravalvular leak <1%; vascular complications <5%; >95% transfemoral access	[11]
Subclinical leaflet thrombosis	Recognition of Hypoattenuated leaflet thickening/Reduced leaflet motion affecting up to 30% of patients	GALILEO, ATLANTIS	Anticoagulation not routinely recommended; clinical significance remains unclear	[65, 66]
Bicuspid aortic valve	From contraindication to routine practice in selected patients	Low Risk Bicuspid Registry, observational studies	Procedural success >95% in Sievers type 0–1; higher pacemaker rates	[67, 68]
Valve-in-valve TAVI	Alternative to redo surgery for failed bioprostheses	PARTNER 2 ViV Registry, Global ViV Registry	Lower mortality than redo-SAVR (2.7% vs. 7.5%); risk of patient-prosthesis mismatch	[69, 70, 71]
Cerebral protection	Embolic protection devices to reduce stroke	PROTECTED TAVR, SENTINEL	Reduced silent brain infarcts; trend toward lower clinical stroke (awaiting definitive data)	[32, 33, 34]

TAVR, transcatheter aortic valve replacement; ViV, valve-in-valve; STS, society of thoracic surgeons.

### 3.4 Paravalvular Leak in TAVI

During SAVR, the diseased valve is completely excised, with the sewing ring and prosthetic sitting within the now vacant annulus. As such, the rates of postoperative paravalvular leaks (PVLs), which are defined by retrograde blood flow between the implanted valve and native tissue [72], are exceedingly low, with some studies reporting 2–10% for all degrees of PVL and 0–4.8% for moderate/severe PVLs [72]. Conversely, TAVI reports much higher PVL rates. Several observational studies have reported at least moderate PVL rates in 10–25% of patients [73], whereas randomized trials have found a PVL rate of 12% post-TAVI. While the literature was previously conflicted about the clinical impact of TAVI, with some purporting PVLs to be a benign finding, and others showing increases in late mortality [74], a meta-analysis of >1500 patients reports that any PVL has a negative impact on outcomes, and is associated with a 2-fold increase in all-cause mortality [75]. While these outcomes may have been acceptable within high-risk patients, in which the alternative would be no surgery at all, these findings raise a fundamental question about whether a 10–25% risk of PVL can be justified in younger, fitter patients when surgical alternatives can offer near-zero rates, at the expense of a more invasive procedure. To compound matters, PVLs have also been shown to be an independent predictor of structural valvular degeneration (SVD) [64], which will require reintervention. Undersized prosthesis, malpositioning, and calcifications have been identified as the most significant predictors of PVLs [76]. Calcification leads to annular asymmetry, resulting in an insufficient seal within the valve and tissue. Similarly, a large, elliptical annulus has also been shown to be a risk factor. Therefore, clinical teams should conduct a thorough preoperative assessment in healthier patients to identify potential predictors of PVL, utilizing multislice CT (MSCT), echocardiographic studies, and hemodynamic studies to identify confounding anatomy. Newer generations of THV can also recapture and reposition the valve, reducing the risk of PVLs. Clinicians should engage in careful discussion with younger patients who are eligible for both procedures, with counseling performed regarding the different complication rates, ensuring patients understand they may be accepting higher long-term morbidity for short-term procedural benefits. Future research on the long-term durability of TAVI should focus on quality-of-life outcomes, paying particular attention to the impact of PVLs on valve longevity and the need for reintervention.

### 3.5 Asymptomatic Severe Aortic Stenosis: From Watchful Waiting to Early Intervention

The evolution of TAVI use in high- to low-risk patients has marked a dramatic expansion of the clinical application of TAVI, raising fundamental questions about the management of aortic stenosis. Asymptomatic aortic stenosis is commonly managed through clinical and echocardiographic follow-ups every 6–12 months, and replacement is recommended only in those with symptoms or severe stenosis that contribute to a reduced left ventricular ejection fraction (<50%) [10]. However, a multitude of trials have suggested that early intervention through TAVI may confer benefits in all-cause mortality and cardiac-related morbidity. Initially, trials compared early SAVR with conservative management and found improved outcomes [77]. Further trials were subsequently performed to investigate early intervention utilizing TAVI, known as the EARLY TAVR trial [78]. Indeed, the EARLY TAVR trial recruited 901 asymptomatic patients aged >65 years with left ventricular ejection fractions (LVEFs) >50% and randomized the patients to either receive clinical surveillance (CS) or early TAVI. These patients were followed for a median of 3.8 years. The primary endpoint was a composite of death from any cause, stroke, or hospitalization for cardiovascular causes. The trial found that the primary composite endpoint was more common in the surveillance group than in TAVI (TAVI: 26.8%; CS: 45.3%; 95% CI: 0.4–0.63; *p* < 0.001) and also found that those in the CS group were more likely to be hospitalized for cardiac reasons, and were associated with worsening left ventricular function. Interestingly, approximately a quarter of the patients assigned to CS underwent valve replacement within the first 6 months, regardless, and the group as a whole reported a reduced quality of life. These findings informed the new 2025 ESC/EACTS guidelines [9], which challenge the previous status quo and recommend early intervention (class IIa A) in asymptomatic patients with severe high-gradient aortic stenosis and preserved LVEF when procedural risk is low. This paradigm shift represents one of the most significant changes in contemporary management of valvular heart disease and reflects the ongoing development of TAVI technology and the current evidence base.

### 3.6 TAVI in Moderate Aortic Stenosis: Prevention of Myocardial Damage

The expansion of TAVI from high- to low-risk patients with severe stenosis has been well-established, yet emerging evidence has shown that intervention while aortic stenosis is still deemed to be moderate may prevent irreversible myocardial damage and improve clinical outcomes. Chronic pressure overload within the left ventricle triggers myocardial remodeling and fibrosis through various pathways, including cardiomyocyte apoptosis, reactive fibrosis, and extracellular matrix disruption [79]. Myocardial fibrosis, assessed by extracellular volume fraction (ECV%) on cardiac MRI, has been demonstrated to be already present in moderate stenosis and independently predicts mortality and hospitalization for heart failure [80]. A multicenter cohort of 457 patients with moderate or asymptomatic severe aortic stenosis found that patients with elevated ECV% (>28.6%) demonstrated significantly worse 5-year event rates (19.3% vs. 9.9%) independent of AS severity [80]. To address the question of whether earlier intervention in patients with moderate stenosis improves outcomes, three major RCTs have either been completed or are in progress [81, 82, 83]. The TAVR UNLOAD [81] trial, including 178 patients, was recently completed and recruited patients with moderate heart failure with reduced ejection fraction (HFrEF) with moderate AS. Patients were randomized to receive TAVI or CS, with the endpoint designated as a composite of all-cause mortality, stroke, disease-related hospitalization, and quality-of-life measures. The TAVI group failed to demonstrate superiority at 23 months; however, TAVI was associated with an increase in quality of life when compared to the CS group. Notably, however, 39% of the CS group progressed to severe stenosis, requiring TAVI, with 20% receiving TAVI within the first year. The authors noted that the trial size may have been underpowered to detect significant differences between the two treatment groups. To address this, the ongoing PROGRESS trial [82] enrolled patients with moderate stenosis and evidence of cardiac damage markers and randomized the patients to receive TAVR with BEVs or to undergo CS. EXPAND TAVR II [83] is an additional ongoing trial that recruited 650 symptomatic patients with moderate stenosis and compares outcomes between TAVR and medical therapy. Until the results of the PROGRESS and EXPAND TAVR II trials are released, current guidelines reserve TAVR for severe stenosis; however, the results of these trials may fundamentally alter our approach to AS management, shifting from valve-centric to a myocardium-centric assessment.

## 4. Valve Technology

As mentioned previously, there are two primary THV systems commonly in use worldwide: the SEVs and BEVs. Mechanical expandable valves (MEVs) have also been trialed and are purported to reduce PVL rates and be advantageous in complex patient anatomy. Despite this, MEVs had a complex delivery system, required extensive operator training, and were recalled from use [84]. Hence, MEVs currently have no clinical indications for use. Both THVs have been extensively validated for clinical use [59, 60], and decision-making relies on operator and center experience, patient anatomy, and co-morbidity considerations.

### 4.1 Balloon-Expandable Valves

Currently, the Sapien 3/Ultra series (Edwards Lifesciences Corporation, Irvine, CA, USA) of BEVs remains the most commonly used BEV in clinical practice [60]. The Sapien 3/Ultra series features an intra-annular trileaflet bovine pericardium valve mounted onto a cobalt–chromium frame. The Sapein 3 (S3) valve also includes the Commander delivery system and eSheath introducer set [85], which reduces the main sheath size from 18.1 Fr to 14.3 Fr. This was thought to reduce major vascular complications [86] compared with the previous iteration of S3, the Sapien XT [87]. The Commander delivery system allows the catheter to flex and navigate complex anatomy and anomalous aortic roots. Furthermore, the S3 features a low frame height and large cell arrangement, allowing the valve to sit below the coronary arteries and facilitate future coronary access [88]. Generally, in the deployment of BEVs, the catheter is passed across the diseased aortic valve, and a balloon is inflated to widen the valve, fracture the calcific deposits within the leaflets, and open the lumen. The stent, which surrounds the crimped valve, expands radially against the aortic root wall. The balloon is then deflated and withdrawn. A polyethylene terephthalate (PET) cuff covers the proximal segment of the stent to create a seal to prevent PVLs. The new valve begins to function immediately. The Sapien X4 is the latest iteration of a BEV; however, this valve is not currently licensed for use. However, the ongoing ALLIANCE [89] trial, as of August 2025, is assessing the efficacy of this valve. The Sapien X4 features RESILIA leaflet tissue [90], an adjustable frame design, and independent valve rotation to address commissural alignment, improve coronary access, and reduce PVL rates.

### 4.2 Self-Expandable Valves

The most commonly used SEVs include the Evolut R/PRO (Medtronic Inc., Minneapolis, MN, USA) and the ACURATE neo (Boston Scientific, Marlborough, MA, USA) [91]. The Evolut R/PRO features trileaflet porcine pericardium valves, mounted suprannally and attached to a nitinol (shape-memory alloy) frame. However, the ACURATE neo utilizes a top-down two-step deployment mechanism [92]. This design is intended to minimize obstruction of the outflow tract during deployment and eliminate the need for rapid ventricular pacing. In contrast to BEVs, SEVs do not require balloon expansion to mount the system frame onto the aortic root wall. Indeed, once the catheter is passed across the annulus and the position is confirmed by TOE or fluoroscopy, the valve is gradually released, allowing repositioning if necessary. Once the correct alignment is confirmed, the full valve is released. The Evolut Pro+ can be repositioned up to three times for optimal positioning. The stent is expanded distal to proximal, with the LVOT side expanding last. This gradual deployment means that rapid ventricular pacing is not required, unlike BEVs.

### 4.3 THV Selection Considerations

As mentioned previously, the decision to use a BEV or SEV depends on a multitude of factors, including annulus size, aortic root geometry, coronary access needs, and operator experience. Each THV has distinct advantages and certain clinical scenarios in which its use is preferred. However, it is also important to consider key disadvantages that may contraindicate the use of certain designs. Due to the rapid deployment of BEVs, rapid ventricular pacing is required, which may cause hemodynamic instability in patients with severely reduced ejection fractions and may worsen clinical outcomes. The risk of annular rupture is highest in BEVs and, thus, would not be suitable for patients with excessively calcified LVOTs [48, 93, 94]. Risk of coronary obstruction is a key risk to consider when using a selective device, and only so much risk can be mitigated with accurate preprocedural planning. Due to the retrievable and readjustable nature of SEVs, these valves may be preferred in patients with low-set coronary ostia; however, SEVs are supra-annular and, thus, have taller frames, which can obstruct the ostia. Alternatively, BEVs have larger cells and lower frame heights, making these valves a wiser device choice for patients with coronary artery disease. Valve durability is also an extremely important consideration when deciding between THV systems. Specifically, smaller patients with smaller annulus sizes may be more prone to prosthesis–patient mismatch (PPM), in which the effective orifice area is too small to meet cardiac demands, resulting in impaired valvular hemodynamics, accelerated valvular degeneration, and increased valvular gradients [95]. The SMART trial [96] was a large-scale RCT that investigated the efficacy of each system in patients with small annulus areas. The trial found that the incidence of moderate-to-severe PPM was lower in SEVs than in BEVs (SEV: 11.2%, BEV: 35.3%; *p* < 0.001) and that SEVs exhibited better valve hemodynamics. This is thought to be due to the supra-annular design common in SEVs. A summary of clinical and haemodynamic outcomes of the respective valve types have been compiled in Table 3 (Ref. [44, 91, 96, 97, 98]).

**Table 3.** **A comprehensive comparison of clinical and hemodynamic outcomes between current-generation balloon-expandable and self-expanding valves based on recent meta-analyses**.

		Balloon-expandable valves (Sapien 3/Ultra)	Self-expanding valves (Evolut R/PRO)	Statistical significance	References
Clinical outcomes					
	30-day mortality	2.5–3.2%	2.8–3.5%	No difference (*p* = 0.56)	[91, 97]
	1-year mortality	8.5–10%	9.0–11%	No difference (*p* = 0.86)	[91, 97]
	Stroke (30-day)	2.5–3.0%	2.8–3.5%	No difference (*p* = 0.97)	[91, 97]
	Annular rupture	0.5–1.0%	<0.1%	Higher with BEV (*p * < 0.01)	[44, 98]
Hemodynamic outcomes					
	Mean gradient (1 year)	12–15 mmHg	7–9 mmHg	Lower with SEV (*p * < 0.001)	[91, 98]
	Effective orifice area	1.5–1.7 cm^2^	1.8–2.1 cm^2^	Larger with SEV (*p * < 0.001)	[91, 98]
	Patient–prosthesis mismatch	25–35%	10–15%	Lower with SEV (*p * < 0.001)	[91, 96, 97]
Complications					
	Moderate/severe PVLs	1–2%	3–5%	Higher with SEVs (OR 1.76; *p* = 0.01)	[91, 97]
	Permanent pacemaker	6–10%	15–25%	Higher with SEVs (OR: 1.57; *p * < 0.001)	[91, 97]
	Major vascular complications	4–5%	5–7%	No difference (*p* = 0.15)	[91, 97]

BEV, balloon-expandable valves; PVLs, paravalvular leaks; SEVs, self-expanding valves; OR, odds ratio.

### 4.4 Special Clinical Scenarios: Bicuspid Valves and Valve-in-Valve

There are two specific common use scenarios in which evidence on the optimal valve choice is conflicting: bicuspid aortic valves and ViV procedures. Bicuspid valve disease is common in younger patients with aortic valve pathology, and the patients are often excluded from large-scale TAVI trials. This is partly due to the unusual anatomy of bicuspid valves, which usually have larger annuli containing elliptical, asymmetrical leaflets, and commonly co-present with dilated aortic roots [67, 99]. As such, a PVL is a common foreseeable complication. While earlier meta-analyses of observational studies suggest that new-generation BEVs were associated with significantly lower rates of PVLs compared to SEVs [67], emerging data indicate that valve selection may be more nuanced. A recent retrospective cohort study of 2553 patients by Li *et al*. [100] demonstrated that SEVs in bicuspid patients were associated with a lower all-cause mortality than BEVs. In addition, these patients also displayed lower mean gradients and larger effective orifice areas. These findings suggest that SEVs conform better to elliptical bicuspid annuli and that improved hemodynamic performance may outweigh concerns about PVL rates. Interestingly, the study also distinguished between Sievers type 1 and type 0 bicuspid patients, with type 0 patients having two valve leaflets and type 1 patients having three leaflets connected by a raphe; moreover, type 0 patients had a better long-term prognosis after TAVI than tricuspid and type 1 patients. These findings highlight the importance of individualized valve selection in patients with varying anatomies, considering immediate procedural outcomes and long-term hemodynamic function.

ViV procedures are also becoming increasingly common. Previous populations of patients who underwent SAVR and TAVR are aging, and SVD rates are increasing as a result. ViV TAVI procedures are a less invasive alternative to redo SAVR and are preferable in high-surgical-risk patients [68]. Recent studies have found no significant differences in complication rates and 1-year mortality, and lower rates of 30-day mortality. However, ViV is associated with higher post-procedure gradients and higher rates of severe PPM [69, 70]. Thus, identifying which THV system provides optimal patient outcomes in the ViV procedure is important. A BEV is beneficial as deployment allows balloon fracture of the surgical bioprosthetic valve to facilitate ViV and increase effective orifice area. TAVI after TAVI also poses a unique clinical question, and some have purported that, to reduce valvular gradients, the most effective method is to use an SEV implant for degenerated BEVs, and vice versa [71]. Currently, there is a distinct paucity of high-quality studies assessing whether a BEV or SEV provides better outcomes in ViV procedures; meanwhile, the Edwards Sapien 3 is the only valve approved by the Food and Drug Administration (FDA) for TAVI-in-TAVI procedures.

## 5. Current Challenges and Future Perspectives in the Modern TAVI Era

As the scope of TAVI use expands to younger and lower-risk patients, clinicians face pertinent clinical questions to optimize outcomes for a population for whom high-quality study data are limited. As TAVI was only officially approved a decade ago, the associated 10–15-year performance in patients remains unknown, and the efficacy and efficiency of TAVI in younger patients remain in question. Younger patients are usually offered a surgical valve replacement with mechanical valves. Many prefer this over tissue valves due to the lower reintervention rates with mechanical valves and the increased complexity of reoperations after TAVI [101]. Despite this, TAVI use has skyrocketed in patients aged under 65 years, and implantation rates have increased dramatically from 7.1% in 2013 to 54.7% in 2021, with more than half of patients aged under 65 years with severe stenosis receiving TAVI [102]. Therefore, further understanding of the durability and performance of TAVI after 10–15 years, and of how to optimize outcomes, is critical to improving patient outcomes in this emerging population.

### 5.1 Structural Valvular Degeneration in TAVI

The definition of SVD has recently been streamlined by the Valve Academic Research Consortium 3 (VARC3) [103] committee to improve data collection and increase the comparability of future studies. Bioprosthetic valve dysfunction may be underpinned by four pathological mechanisms: SVD, such as pannus formation; nonstructural valve dysfunction, such as paravalvular regurgitation or PPM; endocarditis; or thrombosis. VARC additionally defined three stages of SVD in relation to valve function: stage 1, morphological deterioration without hemodynamic effect; stage 2, moderate hemodynamic valve deterioration; stage 3, severe hemodynamic valve deterioration.

Tissue valves are inherently more prone to SVD [95], and a thorough analysis of the clinical outcomes, especially in young patients, is vital for informing clinical decision-making regarding valve choice and operative technique. As the median survival in 65–69 year olds receiving valve replacement is 13 years [104] and multiple registries have reported an incidence of SVD between 4.8% and 13.3%, five to seven years post-TAVI [105, 106, 107], young patients undergoing TAVI will likely need an additional valve procedure in the future. In fact, rates of SVD seem to be inversely correlated with the age of implantation [108]. Some have hypothesized that stronger immune responses to xenografts, coupled with increased opportunities for calcification, contribute to damage to bioprosthetics [65, 101, 109]. Current high-quality evidence on the long-term durability of TAVI compared to SAVR using the latest valve technology is severely lacking; however, the current head-to-head RCTs in high-risk populations show promising results. In the CoreValve-High Risk trial, in which SVD was defined using the European Association of Percutaneous Cardiovascular Interventions (EAPCI) criteria rather than VARC3, the incidence of severe SVD was similar between the TAVI and SAVR groups, and moderate SVD was more common in the SAVR group than in TAVI. The NOTION trials [57] (included low-surgical risk patients and showed an appreciable decrease in the risk of SVD in TAVI compared with SAVR (TAVI: 13.9% vs. SAVR: 28.3%; *p* = 0.0017), but did not show a difference in death due to valve dysfunction. Despite including low-risk patients, the mean age in the NOTION trial was still considerably high at 79 years, and, thus, may have underestimated the true rate of SVD, as some patients may suffer from other complications or causes of mortality before SVD. Presently, there is no distinct head-to-head study on the durability of TAVI compared to SAVR based on the definitions outlined by VARC3 in patients aged <65 years.

When discussing long-term outcomes of TAVR in younger patients, one must also consider the safety of management after TAVR valve failure. Research around this area is critical to support clinical decision-making. Many centers opt for surgical reoperation after early TAVR failure, whether it be due to PVL, SVD, or valve endocarditis. While a necessary procedure, SAVR after the failure of TAVR has been reported to have poor outcomes, with one observational study reporting an operative mortality rate of 17.1% [66]. Authors speculate that neointimal growth is the likely culprit [66], leading to difficulties in extracting the TAVR valve and, thus, a longer cardiopulmonary bypass time when compared to SAVR. A recent study presented at the 2023 American Association for Thoracic Surgery (AATS) convention corroborates this finding that while surgical TAVR reintervention was uncommon [110], operative mortality was far higher (15.8%; *p* = 0.07) when compared to surgical reintervention after ViV-TAVR (0%; *p* = 0.07) [110]. Interestingly, the study found that patients were much more likely to require reintervention after VIV-TAVR; however, reintervention had 0% mortality, whereas redo surgery after TAVR was rare, but associated with higher mortality. These studies reinforce the notion that careful clinical evaluation is critical before TAVR to avoid reoperation or to optimize outcomes after TAVR implantation, as younger TAVR patients are likely to require reintervention in the current era.

While the current body of literature confirms that THVs yield favourable outcomes at 5 years of follow-up, extensive data with longer follow-ups are lacking, especially in younger patients. This represents a critical knowledge gap as the procedure is adopted and expanded toward a younger population. While we await the 10-year follow-up in low-risk trials such as the NOTION and PARTNER3 trials, the durability of THVs in younger patients with diverse physiological and clinical profiles must be studied using standardized definitions of SVD.

### 5.2 Subclinical Leaflet Thrombosis

Subclinical leaflet thrombosis (SLT) affects a relatively large proportion of TAVI patients, with some registries reporting a 30% incidence after 1 year [111], and understanding its prevalence, risk factors, and prevention strategies is integral to optimizing patient outcomes after TAVI. SLT is defined by the VARC3 as the presence of valve leaflet thrombosis and reduced leaflet motion (RLM) detected on validated imaging techniques (multi-detector computed tomography (MDCT) or echocardiography (TOE/TTE)) that is not associated with hemodynamic dysfunction, cardiac symptoms, or thromboembolic events. SLT may escalate toward clinically significant valve thrombosis (CST), causing thromboembolic events such as stroke, transient ischemic attack (TIA), retinal occlusion, etc. [103]. In fact, CST has been shown in a meta-analysis to be associated with an increased risk of stroke (relative risk (RR): 3.17; 95% CI: 1.3–7.72) [103]. Thus, identifying precipitating factors and preventive measures is vital to prevent thromboembolic events, but recent studies have also identified a negative effect on valve durability after TAVI [112].

SLT generally presents as an echogenic leaflet thickening on imaging affecting one, two, or all three leaflets [103]. Current studies have shown that various factors may affect the incidence of SLT: male sex, obesity, hypercoagulable states, reduced LVEF, and a small aortic annulus [113, 114]. Interestingly, atrial fibrillation is a protective factor for SLT, likely because these patients are routinely anticoagulated. This may also contribute to the apparently higher rates of SLT in SAVR than in TAVR [115], as SAVR patients are more frequently placed on anticoagulation therapy after discharge. Despite this, the systematic usage of OACs is not beneficial after TAVI, and the effects of these anticoagulants have been investigated in two large RCTs. The GALILEO trial [116] compared the efficacy of OACs versus antiplatelets for patients with no other indication for anticoagulant therapy and found that while the incidence of SLT was reduced, OACs were associated with increased risk of death and major bleeding complications. The ATLANTIS trial [117] reported similar safety metrics but also observed an increase in the number of noncardiovascular deaths among patients administered OACs compared with those with standard care (vitamin K antagonists/antiplatelets). Currently, both European and American guidelines do not recommend systematically utilizing OACs for the first 3 months following TAVR due to the risk of serious bleeding overriding the risk of thromboembolic events.

If there is evidence of leaflet thrombosis with evidence of hemodynamic valve deterioration (stage 2 or 3), thromboembolic escalation, or new signs of heart failure, treatment with OACs is recommended to resolve clinically significant thrombosis, and studies have shown that OACs are effective in resolving these [118, 119]. However, in the case of SLT, no randomized trials have demonstrated a benefit of treating subclinical leaflet thrombosis with OACs; thus, management decisions are based on an individualized assessment of bleeding versus thrombotic risk.

Ultimately, critical knowledge gaps remain in optimizing SLT management. Large-scale RCTs specifically recruiting patients with isolated SLT and no other OAC indications are urgently needed to determine whether treatment improves clinical outcomes. Additionally, long-term studies examining the impact of SLTs on valve durability and patient survival will be essential for developing evidence-based guidelines regarding treatment indications, anticoagulation regimens, and therapy duration. Until such evidence emerges, clinicians must balance individual patient risk profiles while maintaining vigilant surveillance for SLT progression to clinically significant disease.

### 5.3 Next-Generation Valve Technologies

As TAVI technology continues to evolve, the next frontier focuses on developing valve platforms that combine the hemodynamic excellence of current devices with enhanced durability through novel materials and manufacturing approaches. While current THV bioprosthetic technologies have created demand for novel, non-thrombogenic, durable valve materials that could one day replace bioprosthetic valves in TAVI, the inevitable degradation due to SVD and the need for reintervention have also driven this demand. These are termed polymeric heart valves (PHVs). As mentioned previously, one can expect several complications after a TAVI procedure, with successive iterations of valve technologies aimed at a solution. The risk of a PVL was addressed with anti-PVL skirts, and coronary access was improved with larger mesh stents, among other measures. Nonetheless, numerous complications remain, such as cardiac conduction abnormalities necessitating pacemaker implantation and leaflet thrombosis, which can result in stenosis, thromboembolic events, or shortened valve lifespan. To address some of these, current research efforts are focused on developing polymeric valves that retain the hemodynamic profile of current valve systems while incorporating the antithrombotic properties of bioprosthetics. Newer materials and current devices undergoing *in vitro* testing aim to provide improved damage resistance during crimping and deployment, more predictable degradation profiles, and reduced thrombogenicity.

Meanwhile, the current aims in the development of PHV vary. Moreover, various valve materials have been trialed over time but have fallen short due to manufacturing limitations, hydrodynamic performance, or durability concerns during *in vitro* and *in vivo* testing [120, 121, 122]. However, during this time, polyurethanes have emerged as the most viable class of polymer compounds and have been involved in the majority of the most recent PHV developments. Incorporation of novel nanocomposite materials, such as polyhedral oligomeric silsesquioxanes (POSS-PCU) [123], has enabled products to resist oxidative and hydrolytic degradation and to increase their durability during *in vivo* testing. POSS-PCU has been notably incorporated into the recent Triskele TAVR valve [124], which has been shown to exhibit lower thrombogenicity and greater hemocompatibility than porous polytetrafluoroethylene (PTFE) [125]. Another notable PHV incorporating PCU poly(styrene-b-isobutylene-b-styrene), termed SIBS, represents a novel compound developed by Innovia (Innovia LLC, USA). SIBS has recently been incorporated into the Polynova valve and has demonstrated promising results. During hydrodynamic testing, the Polynova PHV demonstrated higher effective orifice areas (EOAs) than standard bioprosthetic SAVRs (Perimount Magna Ease) and TAVRs (Innovare). The Polynova PHV also showed lower postvalvular gradients and excellent durability, surpassing 400 million cycles in a pulse duplicator system during accelerated wear testing (AWT). Siloxane polyurethane-urea (SiPUU) is an additional material that has gained traction throughout experimental trials. The Foldax Tria valve [126] remains the only PHV tested in humans, albeit surgically, and has been trialled in 14 patients with a 1-year follow-up [125]; two patients died due to valve-related causes. The study maintained hemodynamic performance over the follow-up period and improved New York Heart Association (NYHA) functional class. One patient experienced an acute myocardial infarction, thought to be caused by a thrombus obstructing the right coronary artery, potentially originating from the valve sewing ring. The TAVR version, which must be thinner to accommodate catheter delivery, has completed preclinical studies in sheep, and larger studies are planned to support in-human trials.

A clear knowledge gap in evaluating PHVs for human use poses risks of endocarditis and highlights the role of different polymers. At the time of this writing, there have been no formal studies on the link between endocarditis and PHVs. Notably, PHVs create a non-thrombotic environment by endothelializing the polymer [127]. While, to our knowledge, *in vivo* endothelialization of PHVs has not been directly reported in the literature, the authors theorize that material porosity enables capillary ingrowth across the valve skirt, thereby promoting functional endothelialization. This may increase the risk of microbial attachment and accelerate the formation of septic vegetations. *In vivo* studies investigating the link between various materials and endothelialization will help establish causative factors and inform the formulation of effective polymers suitable for PHV use.

Ultimately, no single “optimal” polymer has been identified, and different manufacturers will likely utilize their own novel polymers. What remains important is the capacity for the polymer to retain its microscopic structure during crimping, redeployment, and cyclic hydrodynamic stress; resistance to calcification; low thrombogenicity; satisfactory EOAs. As novel PHVs are developed, it is paramount that future studies include a broad range of data on durability, rates of thromboembolic events, and symptom resolution to validate the PHVs for use in the coming years.

## 6. Conclusion

Transcatheter aortic valve replacement has undergone a dramatic transformation from Cribier’s experimental procedure in 2002 to the standard of care for aortic stenosis across all surgical risk categories. The evolution from a last-resort intervention for inoperable patients to a procedure now recommended for patients aged over 65 years represents a paradigm shift in cardiovascular medicine that has fundamentally altered the treatment landscape for aortic valve disease.

The robust evidence base supporting TAVI has been established through numerous large-scale RCTs demonstrating non-inferiority or superiority to SAVR across high, intermediate, and low-risk populations. Current THV technology provides excellent hemodynamic performance with acceptable complication rates, supported by advances in delivery systems, imaging guidance, and operator experience. The recent publication of the 2025 ESC/EACTS guidelines, which formally endorse early intervention strategies, reflects the rapid development of the TAVI evidence base. However, the ongoing expansion of applications amplifies the urgency of addressing current knowledge gaps, particularly regarding long-term durability, PVL incidence, and subclinical leaflet thrombosis. PVL incidence is a particularly pressing conundrum, significantly more prevalent following TAVI than with SAVR, occurring in 10–25% of patients, compared with near-zero rates with surgical intervention. While acceptable in high-risk patients where the alternative was no surgery, this complication rate becomes increasingly concerning in younger patients with life expectancies exceeding 20 years. The association between PVLs and accelerated structural valve degeneration compounds these concerns, particularly given the complexity and elevated mortality associated with surgical reintervention after failed TAVI.

Long-term durability data remain critically deficient, particularly for patients aged under 65 years. Current follow-ups extend only to 5–7 years, which is insufficient to assess valve performance over the expected lifespan of younger patients. The inverse relationship between age at implantation and rates of structural valve degeneration suggests that younger patients may face inevitable reintervention, with uncertain outcomes given the technical challenges of explanting transcatheter valves. Studies reporting an operative mortality of 17.1% for surgical TAVI explantation highlight the gravity of these limitations.

Subclinical leaflet thrombosis affects up to 30% of TAVI patients at one year, with unclear implications for valve durability and clinical outcomes. While oral anticoagulation reduces the incidence of SLT, trials such as GALILEO and ATLANTIxS demonstrate increased mortality and bleeding complications, precluding the use of systematic anticoagulation strategies. The lack of evidence-based management protocols for SLT represents a significant knowledge gap requiring targeted investigation.

Next-generation valve technologies, particularly PHVs, offer potential solutions to current limitations by improving durability and reducing thrombogenicity. Meanwhile, materials such as POSS-PCU, SIBS, and SiPUU demonstrate promising preclinical results, with the Foldax Tria valve representing the first PHV tested in humans. The integration of considerations for subclinical leaflet thrombosis with emerging polymeric valve technologies offers a unique perspective that bridges current challenges with future solutions. While the 2025 guidelines simplify antithrombotic management by recommending single antiplatelet therapy, the 30% incidence of subclinical leaflet thrombosis and the associated unclear impact on valve durability highlight the need for innovative valve materials that inherently reduce thrombogenic potential. PHVs, with their promise of enhanced biocompatibility and reduced thrombogenicity, may address these fundamental limitations while providing the durability necessary for younger patient populations now eligible for early intervention under current guidelines. However, extensive validation will be required before clinical implementation, with particular attention to the risk of endocarditis and long-term biocompatibility.

Several research priorities emerge from these limitations. Standardized long-term durability studies using VARC3 definitions are essential, particularly in younger populations. Comparative effectiveness research examining optimal valve selection for specific anatomical scenarios, including bicuspid valves and ViV procedures, remains incomplete. Additionally, randomized trials investigating SLT management and the development of predictive models for complications such as PVL and conduction disturbances are urgently needed.

The success of TAVI in transforming the management of aortic stenosis is undeniable, yet the continued expansion of TAVI must be tempered by recognition of current limitations. As the procedure increasingly targets younger patients with longer life expectancies, the imperative for rigorous long-term studies and technological innovation becomes paramount. Future research must address these knowledge gaps to ensure that the remarkable progress achieved in TAVI technology continues to translate into improved patient outcomes across all age groups and risk categories.

## Abbreviations

AATS, American Association for Thoracic Surgery; ACC, American College of Cardiology; AHA, American Heart Association; AKI, acute kidney injury; AV, atrioventricular; AWT, accelerated wear testing; BAV, balloon aortic valvuloplasty; BEV, balloon-expandable valve; CEP, cerebral embolic protection; CI, confidence interval; CST, clinically significant thrombosis; CT, computed tomography; DW, diffusion-weighted; EAPCI, European Association of Percutaneous Cardiovascular Interventions; EACTS, European Association of Cardiothoracic Surgeons; ECG, electrocardiogram; EOA, effective orifice area; ESC, European Society of Cardiology; EuroSCORE, European System for Cardiac Operative Risk Evaluation; FDA, Food and Drug Administration; HR, hazard ratio; LV, left ventricular; LVEF, left ventricular ejection fraction; LVOT, left ventricular outflow tract; MDCT, multi-detector computed tomography; MEV, mechanical expandable valve; MRI, magnetic resonance imaging; MSCT, multislice computed tomography; NYHA, New York Heart Association; OAC, oral anticoagulant; OR, odds ratio; PET, polyethylene terephthalate; PHV, polymeric heart valve; POSS-PCU, polyhedral oligomeric silsesquioxanes-polycarbonate urethane; PPM, prosthesis patient mismatch; PTFE, polytetrafluoroethylene; PVL, paravalvular leak; RCT, randomized controlled trial; RLM, reduced leaflet motion; RRR, relative risk reduction; SAVR, surgical aortic valve replacement; SEV, self-expandable valve; SIBS, poly(styrene-b-isobutylene-b-styrene); SiPUU, siloxane polyurethane-urea; SLT, subclinical leaflet thrombosis; STS, Society of Thoracic Surgeons; STS-PROM, Society of Thoracic Surgeons Predicted Risk of Mortality; SVD, structural valvular degeneration; TAVI, transcatheter aortic valve implantation; THV, transcatheter heart valve; TIA, transient ischemic attack; TOE, transoesophageal echocardiography; TTE, transthoracic echocardiography; VARC, Valve Academic Research Consortium; ViV, valve-in-valve.
